# Evaluating articles on the content and quality of YouTube videos regarding women’s health: a scoping review

**DOI:** 10.4069/kjwhn.2023.08.19

**Published:** 2023-09-26

**Authors:** Jin Hyeon Kim, Hyun Kyoung Kim

**Affiliations:** 1 Department of Emergency Medical Services, College of Nursing and Health, Kongju National University, Gongju, Korea; 2 Department of Nursing, College of Nursing and Health, Kongju National University, Gongju, Korea

**Keywords:** Information sources, Review, Social networking, Women’s health

## Abstract

**Purpose:**

This scoping review investigated the content and quality of YouTube videos on women’s health.

**Methods:**

A literature search of the Cochrane Library, PubMed, Embase, CINAHL, ERIC, and RISS databases was performed using the keywords “(‘youtube’/exp OR youtube OR ‘social media’/exp OR ‘social media’ OR ((‘social’/exp OR social) AND (‘media’/exp OR media))) AND (‘female health care’ OR ((‘female’/exp OR female) AND (‘health’/exp OR health) AND (‘care’/exp OR care)))” from February 21 to 27, 2023. Peer-reviewed analytic studies in English or Korean that focused on women’s health using YouTube were included.

**Results:**

The review identified 21 articles that covered various themes related to women’s health, such as breast cancer, urinary disease, sexual health, pelvic organ prolapse, the human papillomavirus vaccine, Papanikolaou smears, contraception, women’s health information during the coronavirus disease 2019 pandemic, obstetric epidural anesthesia, and placenta accreta. However, the overall quality of the content was low, inaccurate, unreliable, and misleading.

**Conclusion:**

This scoping review demonstrated that YouTube videos on women’s health covered diverse topics, but the quality of the content needed improvement. More reliable and high-quality videos produced by academic institutes and healthcare professionals specializing in women’s health are needed for social media to be usable as a reliable source of women’s health information. The high number of views and shares received by the videos underscores the importance of providing accurate and reliable information on women’s health.

## Introduction

Social media is a term that refers to websites and social network services that facilitate electronic communication and the exchange of information, ideas, and messages. Examples of social media platforms include YouTube, Twitter, Instagram, Facebook, and TikTok. Among these platforms, YouTube is a popular form of social media due to its ability to deliver information quickly and easily through video [1]. Since anyone can upload videos to YouTube, it has high accessibility and widespread popularity. However, due to the open nature of this platform, there is a proliferation of fake news, and the accuracy and reliability of the information presented can often be low. During the coronavirus disease 2019 (COVID-19) pandemic, the need for health information increased, but social engagement declined, leading many people to prefer online resources such as YouTube over in-person advice from specialists [2]. Even after restrictions due to the pandemic have been lifted, and medical professionals can provide health information in face-to-face settings, most people still search for information on YouTube and other sources on the internet, which can significantly impact their decisions [2,3].

YouTube has become a popular source of health information because it contains a vast amount of information, and YouTube videos can be produced quickly and are easy to share [4]. People often turn to YouTube for medical advice on disease diagnosis and treatment options, as well as for educational information on coping strategies as patients [5].

Women experience health problems and issues throughout their lives, regardless of their location or region. In both Eastern and Western cultures, women are often reluctant to disclose their health problems or seek treatment from obstetricians and gynecologists [6]. As a result, women with health problems may turn to YouTube to obtain health information. However, given that anyone can post information on YouTube, it is crucial to perform reliability appraisals of health information [5]. For this reason, we conducted a review of published papers to investigate the types of information related to women’s health that YouTube viewers access and to evaluate the quality and reliability of the information presented.

A scoping review aims to identify the characteristics and objectives of a study by providing a preliminary assessment. One advantage of conducting a scoping review is that it offers an overview of the latest research, and the results can be rapidly integrated into policies [7]. A disadvantage, however, is that bias may occur because the quality appraisal of research, typically employed in systematic reviews, is not required [7]. To address this limitation, we incorporated quality appraisal into our study.

The objectives of conducting this scoping review were to examine YouTube-related papers concerning women’s health, identify prevalent women’s health-related themes, and evaluate the quality of the videos. Analyzing the themes and quality of women’s health-related YouTube videos can offer evidence for their use as a women’s health nursing intervention, as well as implications for women’s health nursing practice and research. The overall goal of this study was to analyze the subjects and quality of women’s health-related issues featured in YouTube videos. More specifically, this study aimed to examine the topics and content of YouTube videos, along with their effects and outcomes.

## Methods

### Study design

In this study, we carried out a scoping review of the literature to evaluate the content and quality of YouTube videos related to women’s health. The thematic analysis phase followed the review methodology framework of Grant and Booth [7], as well as the Preferred Reporting Items for Systematic Reviews and Meta-Analyses extension for Scoping Reviews (PRISMA ScR) reporting guideline, which outlines the assessment criteria for an evidence-based systematic review [8].

### Review process

The scoping review research process involved the following six steps: (1) identifying the research question; (2) identifying relevant studies; (3) study selection; (4) charting the data; and (5) collating, summarizing, and reporting the results [9].

1) Identifying the research question

The research question of this study was defined as “What is the quality and content of YouTube videos related to women’s health?.”

2) Identifying relevant studies

The inclusion criteria for literature selection were as follows: (1) articles written in English or Korean; (2) articles from peer-reviewed journals; (3) content analysis papers focused on YouTube videos related to women’s health; and (4) descriptive, experimental, and analytical studies. The exclusion criteria were: (1) gray literature such as protocol studies, theses and dissertations for degrees, qualitative research, systematic reviews, working papers, preprints, conference presentations, reports, magazine articles, and letters; and (2) articles lacking full text. Detailed selection criteria were determined based on the research questions outlined in the participant, intervention, comparison outcome, setting, time-study design (PICOST-SD) framework [10].

*Participants*: Women

*Intervention*: Video regarding health care or health problems through YouTube

*Comparison*: Information not delivered through YouTube

*Outcome*: Quality, reliability, content, usefulness, accuracy, and effectiveness

*Setting*: Social media platforms including YouTube

*Time*: Cross-sectional, pre-, post-, pre-post-, or repeated-measures study

*Study design*: Descriptive, correlational, experimental, or content analysis study

3) Study selection

Two researchers (JHK and HKK) independently performed the processes of literature search, extraction, quality assessment, and analysis. From February 21 to 27, 2023, they conducted a literature search using a total of six search engines, which included three core databases such as the Cochrane Library, PubMed, and Embase, and other databases such as Cumulative Index to Nursing and Allied Health Literature (CINAHL) Complete, Education Resources Information Center (ERIC), and Research Information Sharing System (RISS) [11]. The search utilized advanced search techniques, MeSH terms, Emtree (Elsevier’s authoritative life science thesaurus), natural language, synonyms, and Boolean operators. The following search terms were used in each search engine: in Cochrane Library, the search keyword used was “YouTube AND Health care in Title, Abstract, Keyword”; in PubMed, “YouTube* AND Healthcare*” was used; in Embase, “(‘youtube’/exp OR youtube OR ‘social media’/exp OR ‘social media’ OR ((‘social’/exp OR social) AND (‘media’/exp OR media))) AND (‘female health care’ OR ((‘female’/exp OR female) AND (‘health’/exp OR health) AND (‘care’/exp OR care)))” was used; in CINAHL complete, “YouTube AND Health care AND Female” was used; in ERIC, “YouTube AND Female AND nursing health care” was used; and in RISS, “YouTube health” was used. In 2023, a search was conducted for peer-reviewed journal articles written in English or Korean that were fully accessible.

4) Charting the data

**Figure 1.**
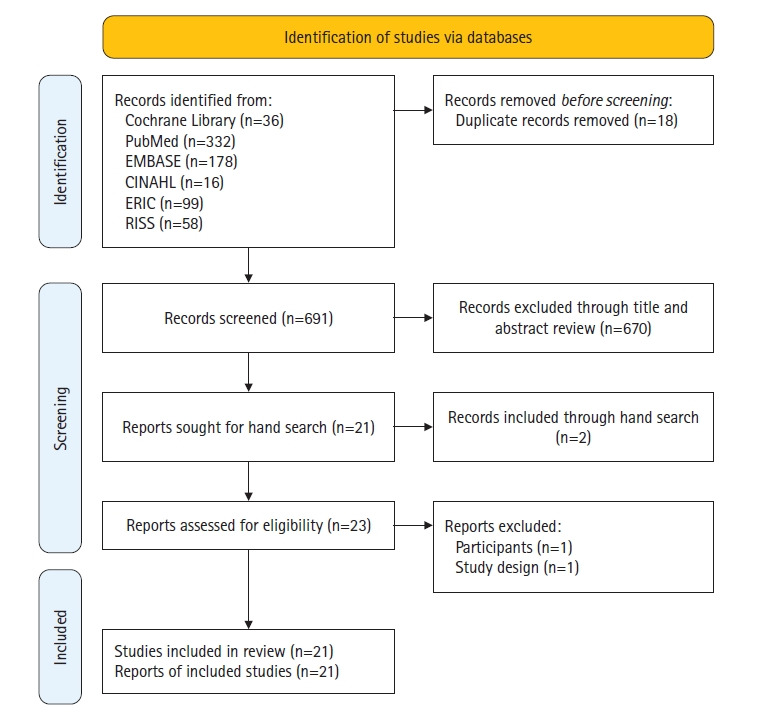
Flow diagram for the literature search.

The search yielded a total of 36 articles published between 2019 and 2023 in Cochrane Library, 322 articles published between 2008 and 2023 in PubMed, 178 articles published between 2009 and 2023 in Embase, 16 articles published between 2012 and 2023 in CINAHL Complete, 99 articles published between 2004 and 2023 in ERIC, and 58 articles published between 2013 and 2022 from RISS. Of the 709 articles initially retrieved, 18 were excluded due to overlapping content: five articles were found in both the Cochrane Library and PubMed, 12 in both the Cochrane Library and Embase, and one in both PubMed and Embase. The titles and abstracts of the remaining articles were thoroughly screened, and those unrelated to women’s health using YouTube were eliminated. This process resulted in 21 articles, including two from the Cochrane Library, 16 from PubMed, none from Embase, two from CINAHL Complete, none from ERIC, and one from RISS. We also conducted a manual search through the reference lists of the articles and identified an additional four relevant articles. Two of these articles were included, bringing the total number of articles to 23. We read the full texts of all 23 articles and selected 21 articles for the final analysis, excluding one article that presented only a simple preference survey and another article that was related to nurses’ health but not to women’s health (Figure 1).

5) Collating, summarizing, and reporting the results

The researchers extracted data independently according to research objectives, produced case reports, and synthesized the data from 21 articles [12-32]. The extracted data items were as follows: first author, publication year, country, content, theme, study design, subjects, number of subjects, evaluators, outcomes, measurement scales, effects, and conclusion. In cases where the extracted data differed between the researchers, the content was harmonized through a meeting.

## Results

### Themes and content of women’s health-related YouTube videos

The articles [12-32] retrieved in this study were published between 2011 and 2023. Turkey had the highest number of publications with nine articles [16,18-23,26,32] followed by the United States with four [12,13,29,31], and Germany with two [25,28]. Additionally, there was one article each from Britain [17], Japan [14], South Korea [27], Saudi Arabia [15], Canada [24], and Italy [30]. The themes covered by the included studies comprised incontinence [12,20,23], breast cancer [18,19], and breast cancer examinations [24], arm exercise after breast cancer surgery [26], *BRCA* mutation test [29], pelvic organ prolapse [28], human papillomavirus (HPV) vaccines [13], Papanicolaou (Pap) smear tests [21], women’s health information [14], female physical examinations [15], contraception [17], pregnancy information during COVID-19 [16], postpartum sexuality [22], obstetric epidural anesthesia [25], placenta accreta [30], sexual education [27], female urethroplasty [32], and female urinary tract infection [31] (one study each). There were 17 content analyses [12,13,15-16,18-20,23-26,28-32], two randomized studies [14,17], and two quasi-experimental studies [22,27]. This study included 18 studies that analyzed English-language YouTube videos [12-15,17,19-26,28-32], as well as three studies [12,28,31] that also analyzed videos shared on other social media platforms, including TikTok, Facebook, Twitter, Instagram, and LinkedIn. Additionally, one study [16] analyzed YouTube videos was in Turkish, and another [27] analyzed videos in Korean. The number of videos analyzed varied from 5 to 4,718, and the number of reviewers ranged from two to 11 (Table 1).

### The effects and outcomes of women’s health-related YouTube videos

The outcome variables used in the included studies comprised content quality [12,18,19,21,23,25,26,30,31], accuracy [16,18,19,31,32], reliability or credibility [21,23,26,30-32], usefulness [15,24,28], view count [14,29], sharing count [14], video length [29], tone [13], source [13], clarity [16], actionability [20], satisfaction [17], acceptance [22], penetration [22], understandability [20], readability [28], education quality [30], sexual knowledge [27], sexual attitudes [27], and parent-child relationships [27]. The measurement tools included DISCERN (a set of quality criteria developed for written consumer health information) [18,19,21,23,26,30,31], self-developed tools [16,22,24,25,28], the Global Quality Score (GQS) [21,26,30,32], Journal of American Medical Association (JAMA) [19,23], view count [14,29], Female Urethroplasty-Specific Checklist Score (FUSCS) [32], National Cancer Development Association (NCDA) [16], medical information content index (MICI) [18], Patient Education Materials Assessment Tool (PEMAT) [20], Alexa score [28], sharing count [14], Video Power Index (VPI), sexual knowledge [27], sexual attitudes [27], parent-child relationships [27], credibility [31], usefulness [12], message tone [13], and source [13].

Informative content related to incontinence was found in 47% of videos on that topic, while commercial content was present in 40.0% [12]. Only 33.0% of videos about the HPV vaccine had a positive tone, whereas a higher percentage had a negative tone [13]. No statistically significant difference was found between the video allocation group and the viewing group in terms of view count and sharing count for women’s health-related videos [14]. In addition, 34.5% of videos related to female physical examinations were found to be useful [15]. Among the videos related to Pap smears, 62.0% had false information. In the videos related to women’s health during COVID-19 pandemic [21], only 4% of the information was accurate despite 40% of the content being clear [16]. There were no statistically significant differences in accuracy and satisfaction related to contraception between the YouTube and non-YouTube viewing groups [17]. Videos related to breast cancer had low average content quality scores of 2.9±1.0 and low accuracy of 5.3±2.8 [18]. Other studies also found that videos related to breast cancer had low average content quality scores of 26.70±10.99 and low accuracy scores of 2.23±0.97 [18]. Videos related to incontinence showed low understandability (57.9±19.8) and low actionability (44.7±35.9) [20]. Postpartum sexuality videos were deemed acceptable by 84% of healthcare professionals and 87% of patients [22]. Incontinence-related videos had an average content quality score of 38.2±11.5 [23]. Only 4.3% of the respondents found videos related to breast cancer examinations to be very useful [24]. A significant portion of epidural anesthesia videos, ranging from 42% to 49%, were deemed inappropriate for not adhering to the aseptic technique [25]. Furthermore, 80% of the videos on arm exercise after breast cancer surgery were found to be useful, but 47.6% contained misleading information [26]. Sexual education videos led to a 36.67-point increase in sexual knowledge scores, and significant increases in scores for sexual attitudes (t=–6.66, *p*<.01) and parent-child attachment (t=–4.40, *p*<.01) were observed [27]. The videos related to pelvic organ prolapse were found to be useful by 73.3% of the respondents. However, the readability of the videos was rated slightly difficult (10.4 points) [28]. The videos related to *BRCA* mutation testing produced by healthcare professionals had a higher number of viewers, with 71 viewers compared to 29 for videos produced by consumers [29]. The quality of content for videos related to placenta accreta was found to be higher in those produced by professionals, with education quality rated at 82.6% and reliability at 26.2%, which were higher than for the videos produced by consumers [30]. Videos related to female urethroplasty produced by universities or hospitals had higher levels of reliability and accuracy compared to those produced by urologists [32]. In addition, the female urinary tract infection videos on YouTube had higher content quality and reliability, but more misleading information, than those on TikTok [31] (Table 2).

## Discussion

This scoping review is a significant contribution as it examined studies that analyzed YouTube videos related to women’s health, identified their content and themes, and analyzed their effects and outcomes. With more people turning to social media platforms for health information, especially during and after the recent pandemic, this review is important because it used analytical studies to evaluate the accuracy, reliability, and quality of women’s health information posted on YouTube [12-32]. The study highlights that videos on various themes related to women’s health have been posted on YouTube, with breast cancer being the most prevalent and popular theme. There was a total of five studies [18,19,24,26,29] on videos related to breast cancer, including two [18,19] on breast cancer itself and one each on gene testing [29], cancer screening [24], and rehabilitation postoperation [26]. The second most prevalent theme was female urinary diseases, which were covered in three studies [12,20,23], including one on urethroplasty [32] and one on urinary tract infection [31]. The third most prevalent theme was sex-related, with two studies [22,27]: one on postpartum sexuality [22] and the other on sex education for girls in upper elementary grades [27]. This study sheds light on the wide range of women’s health topics that are covered on YouTube, indicating significant public interest in these areas. However, the analyses suggest that gynecology diseases are the most frequently discussed topics, with a heavy focus on breast cancer and urinary disorders, while obstetrics-related videos tend to only cover epidural anesthesia [25] and placenta accreta [30]. Thus, this study highlights the need for women’s healthcare professionals to create more gynecology and obstetrics-related videos, which should undergo rigorous analysis and cover a broader range of themes related to women’s health.

The study synthesized the results of previous studies to analyze the effects and outcomes of YouTube videos related to women’s health. The overall quality of YouTube content was low, with low ratings for usefulness, accuracy, and reliability. The study also revealed that videos produced by healthcare professionals were generally of higher quality and contained less fake information than those produced by consumers [23]. Videos produced by academic institutions were also found to have higher quality of content, reliability, and accuracy [32]. Although there has been a strong demand for healthcare information since the COVID-19 pandemic, large amounts of unverified information have spread via the internet, and non-professional videos have potentially had adverse effects. Of the various resources on the internet, those with the most reliable and high-quality information are from research centers and professional societies [33]. Therefore, this study emphasizes the importance of healthcare professionals producing and validating videos, with institutions being preferred producers [32]. Compared to other social media platforms, such as TikTok, Facebook, and Instagram, YouTube was found to have higher quality and reliability of content, indicating that it can be an effective tool for disseminating information related to women’s health [28], as long as accurate information is provided.

Although the quality of YouTube content related to women’s health was generally low [12,18,19,21,25,30] and average level of quality [23], this study found some positive outcomes in the quality of video content. According to the DISCERN total score system, content quality was classified as excellent (63–75), good (51–62), average (39–50), poor (28–38), and very poor (<28). In a systematic review of web-based resources related to complementary and alternative therapy, the DISCERN score averaged 56.13 (standard deviation, 10.25) out of 75 points [34], which was higher than the results of this study. For instance, arm and shoulder exercises after breast cancer surgery posted on YouTube were found to be useful in 80% of cases and considered to be valuable educational tools for preventing musculoskeletal complications due to their high quality and reliability [26]. These findings suggest that YouTube videos can be a helpful tool for providing direct visual assistance and practical guidance, rather than just general knowledge about diseases and conditions. The COVID-19 pandemic has increased the demand for information on pregnancy [16], but the reliability of YouTube videos related to this topic was found to be low. This emphasizes the need for more reliable YouTube videos, especially for vulnerable populations such as pregnant women who may have difficulties obtaining information through face-to-face education during the pandemic [35].

Most YouTube videos related to women’s health focus on changing knowledge rather than skills or attitudes [12-32]. However, in videos that aim to teach techniques, the rate of adherence to aseptic technique was low, ranging from 42% to 47% [25]. The reliability of the procedure was also found to be low, highlighting the need for accurate and reliable videos produced by healthcare professionals. In a study on postpartum sexuality, the experimental group that watched a YouTube video showed a more positive attitude toward sex and felt more comfortable discussing it than the control group that did not watch the video [22]. While most videos have educational purposes to improve knowledge and skills, changing people’s attitudes can also be a useful intervention technique on YouTube. For example, videos with a negative tone about HPV vaccination tend to receive more “likes,” suggesting that viewers may be more drawn to negative aspects of a topic rather than positive ones [13]. Therefore, it is important for both consumers and researchers to be aware of the socio-cultural prevalence of fake news and misleading information on women’s health issues that lack scientific evidence [22].

This study has several limitations. First, there may be themes and effects related to YouTube videos on women’s health that have not been identified since the analyses were conducted indirectly through studies that reviewed these videos. Furthermore, due to the constantly changing nature of YouTube, the results may not accurately reflect the current reality of women’s health-related videos on the platform. Second, only videos produced in English, Korean, and Turkish were included in the analysis, so videos made in other languages were not represented. Third, content analytical and experimental studies were analyzed together, which could lead to problems with comparability.

Nonetheless, this study underscores the importance of producing more high-quality videos on a wider range of topics related to women’s health, utilizing the benefits of prevalence and accessibility offered by YouTube. To ensure the reliability and quality of content, it is recommended that videos be produced by healthcare professionals and that institutions, rather than individuals. Even after the pandemic, YouTube videos are expected to continue to be an important educational resource, as they have become a critical source of information about women’s health in a non-face-to-face society.

## Figures and Tables

**Table 1.** Content and themes of selected studies (N=21)

Study	Publication year	Country	Content and themes	Study design	Subjects	Number of subjects	Evaluators
Sajadi and Goldman [12]	2011	United States	Urinary incontinence	Content analysis	YouTube, Facebook, and Twitter videos in English	30 YouTube	Several healthcare professionals
						30 Facebook	
						30 Twitter	
Briones et al. [13]	2012	United States	HPV vaccine	Content analysis	YouTube videos in English	172 out of 350 YouTube	Three coders
Kiriya et al. [14]	2018	Japan	Women’s health information	Randomized controlled trial	YouTube videos in English	4718 out of 8353 YouTube	Three (obstetrics, gynecology, and midwife)
Abdulghani et al. [15]	2019	Saudi Arabia	Female physical examinations	Content analysis	YouTube videos in English	457 YouTube	Two authors
Gursoy and Peker [16]	2020	Turkey	Pregnancy information during COVID-19	Content analysis	YouTube videos in Turkish	42 out of 100 YouTube	Two gynecologists
Stephenson et al. [17]	2020	Britain	Contraception in young women	Randomized controlled trial	YouTube videos in English	Experimental : 464	11 authors
						Control : 463	
Yuksel and Cakmak [18]	2020	Turkey	Breast cancer	Content analysis	YouTube videos in Turkish	76 out of 133 YouTube	Two doctors
Yurdaisik [19]	2020	Turkey	Breast cancer	Content analysis	YouTube videos in English	50 YouTube	Two professors
Baran and Yilmaz Baran [20]	2021	Turkey	Urinary incontinence	Content analysis	YouTube videos in English	112 out of 150 YouTube	Two (urologist and gynecologist)
Parabhoi et al. [21]	2021	Turkey	Pap smear examinations	Content analysis	YouTube videos in English	200 YouTube	Two physicians
Rosen et al. [22]	2021	Turkey	Postpartum sexuality	Quasi-experimental design	YouTube videos in English	5 YouTube	Advisory team
Salman and Bayar [23]	2021	Turkey	Female incontinence	Content analysis	YouTube videos in English	40 out of 100 YouTube	Two urologists
Brar et al. [24]	2022	Canada	Breast cancer examination	Content analysis	YouTube videos in English	162 out of 200 YouTube	Two authors
Flinspach et al. [25]	2022	Germany	Obstetric epidural anesthesia	Content analysis	YouTube videos in English	16 out of 600 YouTube	11 healthcare professionals
Güloğlu et al. [26]	2022	Turkey	Arm exercises after breast cancer surgery	Content analysis	YouTube videos in English	172 out of 350 YouTube	Two (surgeon and physiotherapist)
Hong [27]	2022	South Korea	Sexual education	Quasi-experimental design	YouTube videos in Korean	9 parent-child pairs	One author
Hüsch et al. [28]	2022	Germany	Pelvic organ prolapse	Content analysis	YouTube, Google, Facebook, LinkedIn, and Instagram videos in English	30 YouTube	Seven authors
Laforet et al. [29]	2022	United States	*BRCA* mutation test	Content analysis	YouTube videos in English	100 YouTube	Four authors
Collà et al. [30]	2022	Italy	Placenta accreta	Content analysis	YouTube videos in English	39 out of 64 YouTube	Two investigators
Tam et al. [31]	2022	United States	Female urinary tract infections	Content analysis	YouTube and TikTok videos in English	50 YouTube	Three urologists
						50 TikTok	
Sahin et al. [32]	2023	Turkey	Female urethroplasty	Content analysis	YouTube videos in English	47 out of 38 YouTube	Two urologists

HPV: human papillomavirus; COVID-19: coronavirus disease 2019.

**Table 2.** Outcomes and effects of selected studies (N=21)

Study	Outcomes	Measurement scales	Effects	Conclusions
Sajadi and Goldman [12]	Content quality	Usefulness	Information: 47.0%	Insufficient useful content
			Commercial: 40.0%	

Briones et al. [13]	Source	Type of source	News: 36.1%	The majority of videos had a negative tone and were disapproving regarding the HPV vaccine
	Tone	Tone of message	Positive tone: 33.0%	
Kiriya et al. [14]	Shares	Share count	Shares: 0.9%/1.1%, *p*=.53	Not effective
	Views	View count	Views: 5.1%/5.3%, *p*=.44	
Abdulghani et al. [15]	Usefulness	Accuracy of knowledge and demonstration	Highly useful: 34.5%	Various uses for medical education
			Useful: 47.7%	
Gursoy and Peker [16]	Content quality	DISCERN	Useful: 37.9%	Misleading information
	Reliability	GQS	Misleading: 62.0%	
Stephenson et al. [17]	Clarity	Developed scale	Clarity: 40.0%	Low-quality and unreliable information
	Accuracy	NCDA	Accuracy: 3.0%	
Yuksel and Cakmak [18]	Effectiveness	Contraception effectiveness	OR, 0.87; 95% CI, 0.60–1.27	No statistically significant difference between the two groups
	Satisfaction	Satisfaction	OR, 0.93; 95% CI, 0.69–1.25	
Yurdaisik [19]	Content quality	DISCERN	2.9±1.0 (1–5)^†^	Low-quality and untrustworthy
	Accuracy	MICI	5.3±2.8 (1–5)^†^	
Baran and Yilmaz Baran [20]	Content quality	DISCERN	26.70±10.99 (15–75)^†^	Poor overall quality
	Accuracy	JAMA	2.23±0.97 (0–4)^†^	
Parabhoi et al. [21]	Understandability	PEMAT	57.9%±19.8%	Not understandable and actionable for users
	Actionability	VPI	44.7±35.9 (0–100)^†^	
Rosen et al. [22]	Acceptance	Developed scale	Healthcare providers: 84%	Acceptable and effective to disseminate evidence
	Penetration		Patients: 87%	
Salman and Bayar [23]	Content quality	DISCERN	38.2±11.5 (15–75)^†^	Average level of quality
	Reliability	JAMA	1.4±0.6 (0–4)^†^	
Brar et al. [24]	Usefulness	Developed scale	Very useful: 4.3%	Necessary to create reliable and useful YouTube videos
			Moderate: 17.9%	
			Somewhat: 39.5%	
			Not useful: 38.3%	
Flinspach et al. [25]	Content quality	Developed scale	Aseptic technique followed: 42%–49%	Unsuitable for self-study due to serious errors
Güloğlu et al. [26]	Content quality	DISCERN	Useful: 80.0%	Important to protect patients from musculoskeletal system complications
	Reliability	GQS	Misleading: 47.6%	
Hong [27]	Sexual knowledge	Sexual knowledge	Increase of 36.67 points	Effective for improving sexual perception and parent-child relationship
	Sexual attitudes	Sexual attitudes	t=–6.66, *p*<.01	
	Parent-child relation	Parent-child relationship	t=–4.40, *p*<.01	
Hüsch et al. [28]	Usefulness	Developed scale	Useful: 73.3%	Valuable content but fairly difficult to read
	Readability	Alexa score	Readability: 10.4	
Laforet et al. [29]	View	View count	Professionals: 71, Consumers: 29	Professional YouTube is positive
	Length	Length (minute)	Professionals: 7.6, Consumers: 7.3	
Collà et al. [30]	Content quality	DISCERN	Professionals: 3, Consumers: 2	Overall content quality is low
	Education quality	PEMAT	Professionals: 82.6%, Consumers: 66.7%	
	Reliability	GQS	Professionals: 26.2%, Consumers: 9.1%	
Tam et al. [31]	Reliability	GQS	Academic: 4, Urologist: 3	Academic videos have more adequate quality and content
	Accuracy	FUSCS	Academic: 36.8%, Urologist: 63.2%	
Sahin et al. [32]	Content quality	DISCERN	YouTube: 5, TikTok: 3, *p*<.001	YouTube is a valuable source
	Credibility	Credibility	YouTube: 5, TikTok: 2, *p*<.001	
	Accuracy	Misinformation	YouTube: 5, TikTok: 3, *p*=.003	

HPV: human papillomavirus; DISCERN: quality criteria for consumer health information; GQS: Global Quality Score; NCDA: National Cancer Development Association; OR: odds ratio; CI: confidence interval; MICI: medical information content index; JAMA: Journal of the American Medical Association; PEMAT: Patient Education Material Assessment Tool; VPI; Video Power Index; FUSCS: Female Urethroplasty-Specific Checklist Score.

† Possible range.
